# Exposure to Air Pollution and Emergency Department Visits During the First Year of Life Among Preterm and Full-term Infants

**DOI:** 10.1001/jamanetworkopen.2023.0262

**Published:** 2023-02-22

**Authors:** Anaïs Teyton, Rebecca J. Baer, Tarik Benmarhnia, Gretchen Bandoli

**Affiliations:** 1 Herbert Wertheim School of Public Health and Human Longevity Science, University of California, San Diego, La Jolla; 2 School of Public Health, San Diego State University, San Diego; 3 Scripps Institution of Oceanography, University of California, San Diego, La Jolla; 4 California Preterm Birth Initiative, University of California, San Francisco, San Francisco; 5 Department of Pediatrics, University of California, San Diego, La Jolla

## Abstract

**Importance:**

Previous studies have focused on exposure to fine particulate matter 2.5 μm or less in diameter (PM_2.5_) and on birth outcome risks; however, few studies have evaluated the health consequences of PM_2.5_ exposure on infants during their first year of life and whether prematurity could exacerbate such risks.

**Objective:**

To assess the association of PM_2.5_ exposure with emergency department (ED) visits during the first year of life and determine whether preterm birth status modifies the association.

**Design, Setting, and Participants:**

This individual-level cohort study used data from the Study of Outcomes in Mothers and Infants cohort, which includes all live-born, singleton deliveries in California. Data from infants’ health records through their first birthday were included. Participants included 2 175 180 infants born between 2014 and 2018, and complete data were included for an analytic sample of 1 983 700 (91.2%). Analysis was conducted from October 2021 to September 2022.

**Exposures:**

Weekly PM_2.5_ exposure at the residential ZIP code at birth was estimated from an ensemble model combining multiple machine learning algorithms and several potentially associated variables.

**Main Outcomes and Measures:**

Main outcomes included the first all-cause ED visit and the first infection- and respiratory-related visits separately. Hypotheses were generated after data collection and prior to analysis. Pooled logistic regression models with a discrete time approach assessed PM_2.5_ exposure and time to ED visits during each week of the first year of life and across the entire year. Preterm birth status, sex, and payment type for delivery were assessed as effect modifiers.

**Results:**

Of the 1 983 700 infants, 979 038 (49.4%) were female, 966 349 (48.7%) were Hispanic, and 142 081 (7.2%) were preterm. Across the first year of life, the odds of an ED visit for any cause were greater among both preterm (AOR, 1.056; 95% CI, 1.048-1.064) and full-term (AOR, 1.051; 95% CI, 1.049-1.053) infants for each 5-μg/m^3^ increase in exposure to PM_2.5_. Elevated odds were also observed for infection-related ED visit (preterm: AOR, 1.035; 95% CI, 1.001-1.069; full-term: AOR, 1.053; 95% CI, 1.044-1.062) and first respiratory-related ED visit (preterm: AOR, 1.080; 95% CI, 1.067-1.093; full-term: AOR,1.065; 95% CI, 1.061-1.069). For both preterm and full-term infants, ages 18 to 23 weeks were associated with the greatest odds of all-cause ED visits (AORs ranged from 1.034; 95% CI, 0.976-1.094 to 1.077; 95% CI, 1.022-1.135).

**Conclusions and Relevance:**

Increasing PM_2.5_ exposure was associated with an increased ED visit risk for both preterm and full-term infants during the first year of life, which may have implications for interventions aimed at minimizing air pollution.

## Key Points

**Question:**

What is the association between fine particulate matter (PM_2.5_) exposure and emergency department (ED) visits during the first year of life, and are preterm infants more susceptible to PM_2.5_ exposure than full-term infants?

**Findings:**

In this cohort study of 1 983 700 infants, a positive association was observed between PM_2.5_ exposure and all-cause, infection-related, and respiratory-related visits. Preterm and full-term infants were most susceptible to having an all-cause ED visit during their fourth and fifth months of life.

**Meaning:**

These findings suggest that increased PM_2.5_ exposure was associated with an increased ED visit risk; thus, strategies aimed at reducing PM_2.5_ exposure for infants may be warranted.

## Teaser

This cohort study examines the association of air pollution exposure and emergency department visits in the first year of life in full-term and preterm infants in California.

## Introduction

Exposure to air pollution, specifically particles measuring 2.5 μm or less in diameter (PM_2.5_), has adverse health effects.^1^ These include aggravated respiratory symptoms, increased susceptibility to infections, worsened asthma, decreased lung function, increased outpatient visits, and hospital admissions for neurodegenerative, cardiopulmonary, and respiratory diseases, and premature death due to cardiovascular, respiratory, and cerebrovascular conditions.^1,2,3,4,5,6,7,8^

Certain individuals are more vulnerable to the health impacts of PM_2.5_, including children.^9,10,11^ Early childhood exposure has been associated with chronic and acute health issues.^12,13,14,15^ Due to having higher breathing rates than adults, children breathe in more pollutants relative to their body size.^13^ Furthermore, children’s lungs, brains, and cardiovascular and immune systems are not fully developed, especially in early life stages, making them more susceptible to inflammation and other damage caused by pollutants.^12,14^ In particular, preterm infants may be at increased risk from air pollution. Their impaired capacity to handle oxidative stress after birth increases their susceptibility to lower air pollution levels compared with full-term infants, thereby exacerbating their postnatal lung function impairment.^16^

Previous studies have focused on PM_2.5_ exposure during pregnancy and birth outcome risks, including preterm birth,^17,18,19,20,21,22,23^ low birth weight,^18,20,21,23,24,25,26^ and stillbirth and late fetal death,^17,21,27^ and certain childhood outcomes, such as respiratory diseases and impaired lung health.^13,28,29,30^ However, to our knowledge, only 2 studies have evaluated the influence of PM_2.5_ exposure in infancy on hospitalization or emergency department (ED) visit risk, with inconsistent results.^31,32^ This association during early life is not often studied, as birth certificates and hospital admissions data are not commonly combined. Currently, only respiratory-related outcomes have been assessed in early life, despite the range of health issues from PM_2.5_ exposure that are not limited to respiratory conditions. Given the knowledge gaps about which organ systems are affected by PM_2.5_ exposure, a larger scope is needed for examined health outcomes. These studies should also be conducted with larger sample sizes to quantify this impact precisely in the population. Susceptibility by preterm birth status has additionally not been considered, nor has effect modification been explored to identify who in the population is most at risk. Lastly, this association has not been assessed at a more refined temporal resolution, which may provide insight about windows of susceptibility. The association of PM_2.5_ exposure on infant health is critical to study, given the consequences that childhood air pollution exposure can have across the life course.^33^ Furthermore, air pollution exposure is modifiable through shifts in behavior and policy implementation, making it possible to reduce its adverse health consequences.

Resulting from this incomplete understanding, more comprehensive assessments are needed. Therefore, this study assessed the association between PM_2.5_ exposure and all- and specific-cause ED visits during the first year of life and examined whether preterm infants are more susceptible to PM_2.5_ exposure compared with full-term infants.

## Methods

### Study Population

This cohort study was approved by the Health and Human Services Agency’s Committee for the Protection of Human Subjects for the State of California and the University of California, San Diego Human Research Protections Program.^34^ This study followed the Strengthening the Reporting of Observational Studies in Epidemiology (STROBE) reporting guideline. Informed consent was waived because of the impracticality of collecting consent and the research had minimal risk to the participants.

The Study of Outcomes in Mothers and Infants (SOMI) is a population-based administrative cohort study comprised of birth certificates from all California births between 2005 to 2019.^34^ Birth certificates were linked to hospital discharge records maintained by the California Office of Statewide Health Planning and Development, hereafter referred to as health records.^34^ These health records were linked from 1 year prior to the infant’s birth through 1 year after birth, resulting in a cohort of more than 6 million mother-child pairs.^34^

This study included live-born, singleton deliveries between January 1, 2014, and December 31, 2018. Because a complete case analysis was conducted, participants with missing data related to the exposure, covariates, or outcome were excluded (n = 191 480). Infants with a non-California residential ZIP code and those who remained at the hospital for more than 20 weeks between their birth date and discharge date from the birth admission were also excluded (eFigure 1, eTable 1, and eTable 2 in Supplement 1). The final analytic sample consisted of 1 983 700 births (91.2%).

### Exposure

PM_2.5_ exposure was obtained from Aguilera et al^35^ using an ensemble model that integrated several machine learning algorithms (eg, random forest, gradient boosting, and deep learning) and a range of variables. These variables included daily PM_2.5_ measurements from the Environmental Protection Agency Air Quality System, aerosol optical depth, meteorological variables, land use variables, and smoke plumes.^35^ From this model, daily PM_2.5_ measurements (μg/m^3^) were estimated at the ZIP code-level across California from 2006 to 2019.^35^ For this study, daily PM_2.5_ exposure from 2014 to 2019 was averaged at the weekly level, where week 1 began on January 1, 2014, based on the infant’s residential address at birth.

### Outcome

The first all-cause ED visit for each infant’s first year of life was collected from health records. For those with an ED visit, the week during which the visit occurred was obtained. The first specific-cause ED visits were then examined, including infections and respiratory conditions, separately. These causes were selected due to existing evidence regarding increased infection susceptibility and respiratory system consequences from PM_2.5_ exposure.^1,5,36,37^ Both *International Classification of Diseases, Ninth Revision *(*ICD-9*) and *International Statistical Classification of Diseases and Related Health Problems, Tenth Revision *(*ICD-10*) codes were used to identify specific ED causes (eTable 3 in Supplement 1). For each specific-cause visit analysis, individuals with ED visits other than the one of interest were excluded due to competing risks.

### Covariates

The existing literature and a directed acyclic graph were used to identify potential confounders, including payment type for delivery (private insurance coverage, Medi-Cal, self-pay, other, or unknown payment), parity (nulliparous or multiparous), education (less than 12 years or 12 or more years), self-reported race and ethnicity (Hispanic, non-Hispanic Asian, non-Hispanic Black, non-Hispanic White, other, or unknown race), season of birth (2014 to 2018 winter, spring, summer, or fall), and temperature. Race and ethnicity were included as potential confounders due to the existing literature on air pollution and health outcomes, which has shown racial and ethnic disparities in air pollution exposure and health outcomes.^38^ Except for temperature, confounders were extracted or derived from birth records and/or health records. The population-weighted centroids of California ZIP codes were used to extract 2014 to 2019 minimum and maximum daily temperatures (Celsius) from the high spatial resolution (4 km) Gridded Surface Meteorological (gridMET) data set.^39^ Weekly mean temperature ([T_min _+ T_max_]/2) was calculated based on the infant’s residential ZIP code at birth.

### Statistical Analysis

A discrete time approach with pooled logistic regression models was used to assess the association between PM_2.5_ exposure and time to ED visits for each week of the first year of life. Potential spatial clustering for ED visits was considered and a random effect for the intercept on the ZIP code was included in the models. This survival model is advantageous as it quantifies the weekly association between PM_2.5_ exposure and ED visits, allowing for windows of susceptibility to be identified. Individuals were left-censored from birth admission until discharge and followed through the first ED admission or their first 52 weeks of life, whichever came first. Week since birth, payment type for delivery, seasonality, parity, education, race and ethnicity, and time-varying temperature were adjusted for. These models were run for preterm and full-term infants separately and a Cochran Q test of heterogeneity was conducted to assess for effect modification (eTable 4 in Supplement 1). Preterm births were defined as being born before 37 weeks of gestation as noted on birth records. Effect modification by infant sex and payment type for delivery were additionally examined. Weekly specific and pooled (over the first year of life) adjusted odds ratios (AORs) and 95% CIs were examined and deemed significant if the 95% CI did not cross the null or if one of the tails narrowly crossed the null. Analysis was conducted from October 2021 to September 2022 using Stata (StataCorp) and R (R Project for Statistical Computing).

## Results

Of the 1 983 700 infants, 979 038 (49.4%) were female, 966 349 (48.7%) were Hispanic, and 142 081 (7.2%) were preterm (Table 1). Mean PM_2.5_ exposure varied across the years of interest and participants’ mean PM_2.5_ exposure during time at risk ranged from 0.98 to 149.1 μg/m^3^ (eFigure 2 in Supplement 1). A greater proportion of infants in the highest PM_2.5_ exposure quartile compared with lower quartiles were preterm (Q1, 34 304 of 495 923 [6.9%]; Q4, 37 613 of 495 936 [7.6%]), were exposed to higher temperatures (mean [SD], Q1, 16.3 °C [2.7 °C]; Q4, 19.1 °C [2.8 °C]), had mothers who were Hispanic (Q1, 198 339 of 495 923 [40.0%]; Q4, 295 359 of 495 936 [59.6%]), had Medi-Cal coverage (Q1, 204 121 of 495 923 [41.2%]; Q4, 271 198 of 495 936 [54.7%]), had less than a high school education (Q1, 71 873 of 495 923 [14.5%]; Q4, 91 918 of 495 936 [18.5%]), and were multiparous (Q1, 306 135 of 1 236 317 [61.7%]; Q4, 317 594 of 1 236 317 [64.0%]) (Table 1). During the first year of life, 590 888 of 1 983 700 (29.8%) of infants had an ED visit.

**Table 1.** Characteristics of Total Participants Born From 2014-2018 (n = 1 983 700) by Average PM_2.5_ Exposure Quartiles^a^

Characteristics	No. (%)				
	Total (n = 1 983 700)	Quartile 1 (n = 495 923)^b^	Quartile 2 (n = 495 928)^c^	Quartile 3 (n = 495 913)^d^	Quartile 4 (n = 495 936)^e^
Preterm birth	142 081 (7.2)	34 304 (6.9)	33 441 (6.7)	36 723 (7.4)	37 613 (7.6)
Race and ethnicity					
Hispanic	966 349 (48.7)	198 339 (40.0)	206 958 (41.7)	265 693 (53.6)	295 359 (59.6)
Non-Hispanic					
Asian	311 648 (15.7)	79 057 (15.9)	87 342 (17.6)	68 502 (13.8)	76 747 (15.5)
Black	95 889 (4.8)	21 858 (4.4)	19 256 (3.9)	30 427 (6.1)	24 348 (4.9)
White	541 841 (27.3)	174 776 (35.2)	163 148 (32.9)	116 213 (23.4)	87 704 (17.7)
Other/unknown^f^	67 973 (3.4)	21 893 (4.4)	19 224 (3.9)	15 078 (3.0)	11 778 (2.4)
Female	979 038 (49.4)	244 184 (49.2)	244 845 (49.4)	245 921 (49.6)	244 088 (49.2)
Male	1 004 660 (50.6)	251 742 (50.8)	251 080 (50.6)	249 999 (50.4)	251 839 (50.8)
Payment type for delivery					
Private insurance coverage	971 056 (49.0)	270 725 (54.6)	279 000 (56.3)	235 803 (47.6)	185 528 (37.4)
Medi-Cal coverage	882 170 (44.5)	204 121 (41.2)	180 381 (36.4)	226 470 (45.7)	271 198 (54.7)
Self-pay	68 426 (3.5)	4911 (1.0)	19 799 (4.0)	15 025 (3.0)	28 691 (5.8)
Other or unknown payment	62 048 (3.1)	16 166 (3.3)	16 748 (3.4)	18 615 (3.8)	10 519 (2.1)
Less than a high school education	300 204 (15.1)	71 873 (14.5)	57 306 (11.6)	79 107 (16.0)	91 918 (18.5)
Nulliparous	747 383 (37.7)	189 790 (38.3)	193 856 (39.1)	185 407 (37.4)	178 330 (36.0)
Average temperature, mean (SD), °C	18.2 (2.8)	16.3 (2.7)	18.2 (18.2)	19.0 (2.2)	19.1 (2.8)

Abbreviation: PM_2.5_, particulate matter with diameter of 2.5 μm or less.

^a^ Mean (SD) PM_2.5_ exposure for total population: 10.55 (2.86). Values are frequencies and column percentages for categorical variables, and means and standard deviations for continuous variables.

^b^ Quartile 1: 0.98-8.66 μg/m^3^.

^c^ Quartile 2: 8.67-10.43 μg/m^3^.

^d^ Quartile 3: 10.44-12.16 μg/m^3^.

^e^ Quartile 4: 12.17-149.07 μg/m^3^.

^f^ Other or unknown race or ethnicity includes those who are American Indian or Alaska Native, Native Hawaiian or Pacific Islander, 2 or more races, other race, and unknown or refused to state their race.

**Figure 1.**
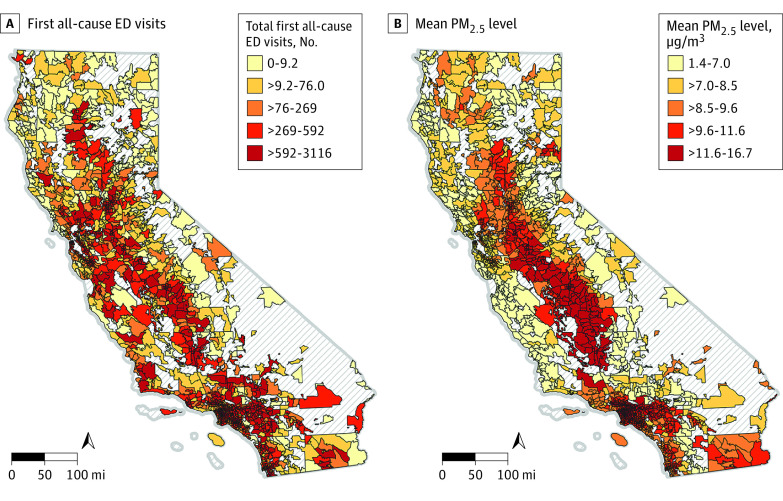
All-Cause ED Visits and Average PM_2.5_ Exposure Maps Maps depicting the total number of first all-cause ED visits for infants born between 2014 and 2018 during their first year of life (A) and mean PM_2.5_ exposure in μg/m^3^ during time at risk for infants (B) at the ZIP code level. Gradient of yellow to red depicts quintiles of increasing severity, and gray hatching depicts ZIP codes for which there was no data. ED indicates emergency department; PM_2.5_, particulate matter with diameter of 2.5 μm or less.

Maps of the total first all-cause ED visits and mean PM_2.5_ exposure at the ZIP code-level are depicted in Figure 1. More spatial variation can be seen for the ED visits, where higher totals were observed along the southern, central, and Bay Area coastline and the Central Valley. In contrast, the highest quintile of mean PM_2.5_ exposure was densely concentrated in the Central Valley and Los Angeles County.

The AORs quantifying the association of PM_2.5_ exposure (increments of 5 μg/m^3^) with ED visits across the entire first year of life are shown in Table 2. Exposure to PM_2.5_ was associated with increased all-cause ED visits for preterm (AOR, 1.056; 95% CI, 1.048-1.064) and full-term infants (AOR, 1.051; 95% CI, 1.049-1.053). Males (AOR, 1.052; 95% CI, 1.049-1.055) and females (AOR, 1.051; 95% CI, 1.048-1.054) had similar odds of an ED visit. Associations were found for infants with self-paid delivery (AOR, 1.086; 95% CI, 1.060-1.114) and for those with Medi-Cal (AOR, 1.049; 95% CI, 1.046-1.052), although the strength of these associations varied. Preterm infants were significantly more at risk than full-term infants regarding respiratory-related visits (preterm: AOR, 1.080; 95% CI, 1.067-1.093; full-term: AOR, 1.065; 95% CI, 1.061-1.069). In contrast, full-term infants were more at risk than preterm infants for infection-related visits (preterm: AOR, 1.035; 95% CI, 1.001-1.069; full-term: AOR, 1.053; 95% CI, 1.044-1.062); however, this difference was not significant.

**Table 2.** Models for PM_2.5_ Exposure and All-Cause and Specific-Cause ED Visits Across the First Year of Life^a^^,^^b^

Population	Adjusted odds ratio (95% CI)	E-Value^c^
**First all-cause ED visit**		
Preterm infants only	1.056 (1.048-1.064)	1.195
Term infants only	1.051 (1.049-1.053)	1.186
Female infants only	1.051 (1.048-1.054)	1.186
Male infants only	1.052 (1.049-1.055)	1.188
Those with private insurance for delivery payment only	1.054 (1.050-1057)	1.191
Those with Medi-Cal for delivery payment only	1.049 (1.046-1.052)	1.182
Those who self-paid for delivery only	1.086 (1.060-1.114)	1.252
Those with other/unknown payment type for delivery only	1.077 (1.062-1.093)	1.236
**First infection-related ED visit**		
Preterm infants only	1.035 (1.001-1.069)	1.149
Term infants only	1.053 (1.044-1.062)	1.189
**First respiratory-related ED visit**		
Preterm infants only	1.080 (1.067-1.093)	1.241
Term infants only	1.065 (1.061-1.069)	1.214

Abbreviation: ED, emergency department.

^a^ PM_2.5_ exposure is included in increments of 5 μg/m^3^.

^b^ Results from Cochran Q test to compare subgroup heterogeneity can be found in eTable 3 in Supplement 1. Results shown separately by preterm birth status, sex, and payment type for delivery. Models stratified by preterm birth status and sex are adjusted for week since birth, payment type for delivery, parity, education, race and ethnicity, seasonality, and time-varying temperature. Models stratified by payment type for delivery are adjusted for week since birth, parity, education, race and ethnicity, seasonality, and time-varying temperature.

^c^ The e-value quantifies the strength of an unmeasured confounder to explain away the observed association.

**Figure 2.**
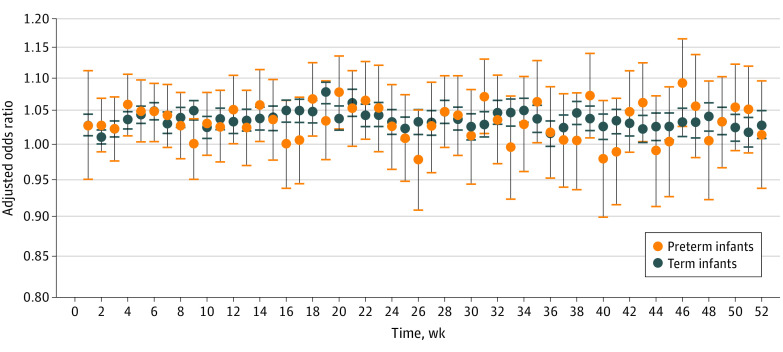
Weekly Association Between PM_2.5_ Exposure and First All-Cause ED Visits for Preterm and Full-term Infants Models using a discrete time approach to assess the association between PM_2.5_ exposure (increments of 5 μg/m^3^) and time to first all-cause ED visits during each week of the first year of life. The orange circles indicate the adjusted odds ratios for preterm infants, the blue circles indicate the adjusted odds ratios for full-term infants, and the error bars indicate 95% CIs. Models were adjusted for payment type for delivery, parity, education, race and ethnicity, seasonality, and time-varying temperature. The y-axis is provided on the logarithmic scale. ED indicates emergency department; PM_2.5_, particulate matter 2.5.

Figure 2 provides AORs and 95% CIs assessing PM_2.5_ exposure (increments of 5 μg/m^3^) and time to first all-cause ED visits during each week of the first year of life stratified by preterm birth status (eTable 5 and eFigure 3 in Supplement 1). AORs were higher though more varied for preterm than full-term infants. Ages 18 to 23 weeks were associated with the greatest risk of all-cause ED visit in general (range of AORs, 1.034; 95% CI, 0.976-1.094 to 1.077; 95% CI, 1.022-1.135), with 95% CIs mostly above the null for preterm infants. In contrast, full-term infants had attenuated positive AORs but precise CIs above the null across most weeks. Full-term infants were most susceptible during weeks 16 to 23 (range of AORs, 1.037; 95% CI, 1.019-1.056 to 1.076; 95% CI, 1.060-1.-93).

**Figure 3.**
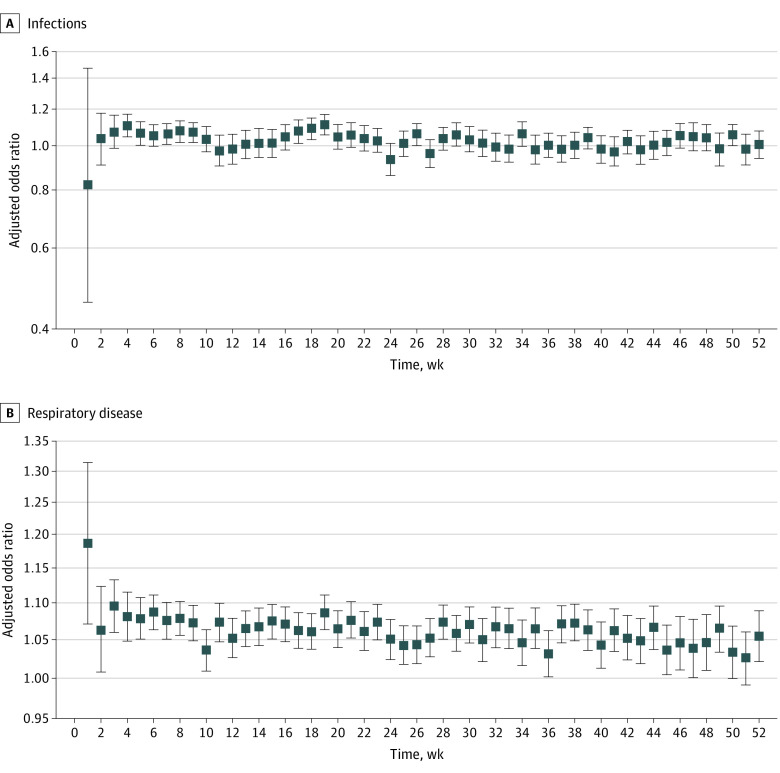
Weekly Association Between PM_2.5_ Exposure and First Infection- and Respiratory-Related ED Visits for the Total Population Models using a discrete time approach assessing the association between PM_2.5_ exposure (increments of 5 μg/m^3^) and time to first specific-cause ED visits, including infections (A) and respiratory diseases (B), during each week of the first year of life, where adjusted odds ratios and 95% CIs are provided for the total population. Models were adjusted for payment type for delivery, parity, education, race and ethnicity, seasonality, and time-varying temperature. The y-axes are provided on the logarithmic scale. The boxes indicate the adjusted odds ratio and the error bars indicate the 95% CIs.

Figure 3 provides weekly AORs for the specific-cause visits in the total population (eFigure4 and eTable 6 in Supplement 1). High infection-related AORs were observed during weeks 17 to 19 for the total population and full-term infants, although most AORs fluctuated close to the null. While higher AORs were observed for later weeks in preterm infants (weeks 29, 39, 46-47), a consistent trend was not observed. In contrast, respiratory-related visits tended to be consistently positive and precise across the year for the total population and for full-term infants. Preterm infants had higher AORs and less precise 95% CIs across the year, with highest AORs in the first 6 months.

eFigures 5 and 6 in Supplement 1 provide weekly all-cause AORs stratified by sex and delivery payment type, respectively (eTable 7in Supplement 1). A similar window of susceptibility can be identified for males and females during weeks 18 to 22, where males have a higher risk during this period than females. Those with private insurance and Medi-Cal had precise 95% CIs and a window of susceptibility for weeks 16 to 24, where those with private insurance had a higher risk during this window. In comparison, those who self-paid for the delivery and those with another or unknown payment type had higher AORs with less precise 95% CIs. A susceptibility window may be seen for those who self-paid during weeks 14 to 23.

## Discussion

Given preterm infant’s potential susceptibility to air pollution, this study investigated the association between PM_2.5_ exposure and ED visits in the first year of life for preterm and full-term infants. While significant differences in risk between preterm and full-term infants were not identified for all-cause visits, greater PM_2.5_ exposure was associated with increased odds of all-cause ED visits during the first year of life for both preterm and full-term infants born in California from 2014 to 2018. No consistent trend in susceptibility with increasing age was identified for these infants; rather, preterm and full-term infants were most susceptible to having an ED visit during the fourth and fifth months of life. Male and female infants were also most susceptible during this window; however, significant effect modification by sex was not identified. In contrast, significant modification was observed by delivery payment type, whereby those who self-paid or had another payment type were most at risk. It is possible that there may be underlying cofactors present in these 2 subgroups that led them to being more susceptible (eg, poverty). Lastly, effects were most pronounced when assessing respiratory-related visits, where preterm infants were significantly more at-risk than full-term infants. Full-term infants were consistently at risk for respiratory-related visits across the first year, and preterm infants were most at risk in the earlier months of life.

Results of previous studies related to infants’ vulnerability to PM_2.5_ exposure have been inconsistent. Karr et al^31^ identified an increased risk between mean PM_2.5_ exposure and bronchiolitis hospitalization for infants during the first year of life in the Puget Sound Region, Washington State. In contrast, Darrow et al^32^ found slight negative associations between PM_2.5_ exposure and ED visits for bronchiolitis or bronchitis and pneumonia, and a positive association for upper respiratory infection visits for infants aged 0 to 1 years in Atlanta, Georgia. This study identified a positive association between PM_2.5_ exposure and respiratory-related ED visits.

Differences exist between the previous studies and this study. Karr et al^31^ and Darrow et al^32^ assessed respiratory-specific outcomes, whereas this study assessed all-cause, respiratory-related, and infection-related visits. Karr et al^31^ assessed hospitalizations, whereas Darrow et al^32^ and the present study assessed ED visits. Darrow et al^32^ averaged PM_2.5_ measurements from monitoring stations using population weighting, and Karr et al^31^ used PM_2.5_ measurements from the monitoring station most proximal to the child’s residential address, whereas this study used average PM_2.5_ concentrations from spatially and temporally resolved air pollution models interpolated at the infants’ residential ZIP codes.

It is critical to assess the association of PM_2.5_ exposure and ED visits in young children and to identify susceptibility windows and exacerbating factors, as these health consequences may carry on through adulthood. ED visit risk increased with increasing PM_2.5_ exposure for all infants, making it important to identify interventions aimed at reducing PM_2.5_ exposure in early childhood. Interventions may include behavior modification such as removing and controlling indoor sources, ventilating the home on days when outdoor pollution is low, and filtering the air,^40^ and policy implementation including land use planning policies, traffic emission mitigation, clean transportation alternatives, adoption of WHO clean air targets, clean production techniques, and pollution-related industry closures.^13,31,41,42,43^

This study had several strengths. To our knowledge, this was one of the first studies to assess the association between PM_2.5_ exposure and both all-cause and specific-cause ED visits in infants. Furthermore, previous studies have solely examined the pooled risk across the first year of life, whereas weekly associations during the first year of life were also assessed to identify potential windows of susceptibility. This study was also particularly advantageous due to its large sample size (n = 1 983 700) and generalizability to California infants. Additionally, PM_2.5_ exposure data was aggregated using the methods from Aguilera et al^35^, who precisely predicted daily PM_2.5_ exposure at the ZIP code-level. This technique considers the spatial heterogeneity of air pollution. Lastly, PM_2.5_ exposure on ED visits was examined by preterm birth status, sex, and delivery payment type, providing insight into which infants are most vulnerable to PM_2.5_ exposure.

### Limitations

This study had limitations. It is possible that not all potential confounders were adjusted for (eg, maternal smoking status). Moreover, payment type for delivery was used as a proxy for socioeconomic status, making residual confounding possible. Table 2 provides e-values, which quantify the strength of an unmeasured confounder to explain away the observed associations.^44^ However, given the large size of these e-values, we are not aware of the existence of such unmeasured confounders that may plausibly reach this level of association with weekly PM_2.5_ concentrations and ED visits. Additionally, infants were left censored from birth to hospital discharge date, as these infants were already at the hospital and therefore not at risk of being admitted to the ED during this time. Nevertheless, it is possible that these infants were impacted by PM_2.5_ exposure while still hospitalized, especially preterm infants, who are more likely to remain in the hospital for a longer duration after birth. This would have attenuated our results and introduced a conservative bias. Lastly, data for temperature and PM_2.5_ exposure were collected for the residential ZIP code at birth, and the possibility of infants moving outside of this ZIP code in their first year of life was not accounted for. While the use of residential ZIP codes is a more refined spatial scale than has often been used in previous studies, this stationary measure does not consider the contribution of mobility (eg, going to nursery care, a park, a grocery store) or moving to a different residential ZIP code to differential PM_2.5_ exposure, leading to potential misclassification.

## Conclusions

These findings suggest that higher PM_2.5_ exposure was associated with an increased risk of ED visits during the first year of life for both preterm and full-term infants. Future studies may wish to incorporate dynamic measures of air pollution, explore additional specific-cause ED visits or outpatient visits, focus on susceptibility by varying degrees of prematurity, investigate effect modification of this association by other factors, consider different sources of PM_2.5_ exposure (eg, wildfire vs nonwildfire PM_2.5_ exposure), examine why windows of susceptibility may exist in the first year of life, and assess the effectiveness of potential interventions in reducing health impacts for children. By understanding who is most at risk and when windows of susceptibility occur, strategies to modify air pollution exposure may be implemented to reduce this health burden.

## Notes

Supplement 1. **eTable 1.** Descriptive Statistics Before and After Excluding Infants With More Than 20 Weeks Between Birth and Discharge Dates **eTable 2.** Differential Exposure and Outcome by Complete Case Analysis and Total Population Including Individuals With Missing Covariates **eTable 3.** Frequency of First Specific-Cause ED Visit for Total Population and Stratified by Preterm Birth Status **eTable 4.** Results from Cochran Q Test **eTable 5.** Weekly Association Between PM_2.5 _Exposure and First All-Cause ED Visits for the Total Population and Stratified by Preterm Birth Status **eTable 6.** Weekly Association Between PM_2.5 _Exposure and First Infection- and Respiratory-Related ED Visits for the Total Population and Stratified by Preterm Birth Status **eTable 7.** Weekly Association Between PM_2.5 _Exposure and First All-Cause ED Stratified by Sex and Payment Type for Delivery **eFigure 1.** Inclusion and Exclusion Criteria Flowchart **eFigure 2. **2014-2019 Timeseries of Average Weekly PM_2.5 _Concentration **eFigure 3.** Weekly Association Between PM2.5 Exposure and First All-Cause ED Visits for the Total Population **eFigure 4.** Weekly Association Between PM2.5 Exposure and First Infection- and Respiratory-Related ED Visits Stratified by Preterm Birth Status **eFigure 5.** Weekly Association Between PM2.5 Exposure and First All-Cause ED Visits for Male and Female Infants **eFigure 6**. Weekly Association Between PM2.5 Exposure and First All-Cause ED Visits Stratified by Payment Type for Delivery

Supplement 2.
Data Sharing Statement
