# Supplementary material for: Exposure to Air Pollution and Emergency Department Visits During the First Year of Life Among Preterm and Full-term Infants
Source: JAMA Netw Open. 2023 Feb 22;6(2):e230262. doi: 10.1001/jamanetworkopen.2023.0262 (PMC9947725; doi:10.1001/jamanetworkopen.2023.0262)
Supplement: Supplement 1. — eTable 1. Descriptive Statistics Before and After Excluding Infants With More Than 20 Weeks Between Birth and Discharge Dates eTable 2. Differential Exposure and Outcome by Complete Case Analysis and Total Population Including Individuals With Missing Covariates eTable 3. Frequency of First Specific-Cause ED Visit for Total Population and Stratified by Preterm Birth Status eTable 4. Results from Cochran Q Test eTable 5. Weekly Association Between PM2.5 Exposure and First All-Cause ED Visits for the Total Population and Stratified by Preterm Birth Status eTable 6. Weekly Association Between PM2.5 Exposure and First Infection- and Respiratory-Related ED Visits for the Total Population and Stratified by Preterm Birth Status eTable 7. Weekly Association Between PM2.5 Exposure and First All-Cause ED Stratified by Sex and Payment Type for Delivery eFigure 1. Inclusion and Exclusion Criteria Flowchart eFigure 2. 2014-2019 Timeseries of Average Weekly PM2.5 Concentration eFigure 3. Weekly Association Between PM2.5 Exposure and First All-Cause ED Visits for the Total Population eFigure 4. Weekly Association Between PM2.5 Exposure and First Infection- and Respiratory-Related ED Visits Stratified by Preterm Birth Status eFigure 5. Weekly Association Between PM2.5 Exposure and First All-Cause ED Visits for Male and Female Infants eFigure 6. Weekly Association Between PM2.5 Exposure and First All-Cause ED Visits Stratified by Payment Type for Delivery [file jamanetwopen-e230262-s001.pdf]

Supplemental Online Content

Teyton A, Baer RJ, Benmarhnia T, Bandoli G. Exposure to air pollution and emergency department visits during the first year of life among preterm and full-term infants. *JAMA Netw Open*. 2023;6(2):e230262. doi:10.1001/jamanetworkopen.2023.0262

eTable 1. Descriptive Statistics Before and After Excluding Infants With More Than 20 Weeks Between Birth and Discharge Dates
eTable 2. Differential Exposure and Outcome by Complete Case Analysis and Total Population Including Individuals With Missing Covariates
eTable 3. Frequency of First Specific-Cause ED Visit for Total Population and Stratified by Preterm Birth Status
eTable 4. Results from Cochran Q Test
eTable 5. Weekly Association Between $PM_{2.5}$ Exposure and First All-Cause ED Visits for the Total Population and Stratified by Preterm Birth Status
eTable 6. Weekly Association Between $PM_{2.5}$ Exposure and First Infection- and Respiratory-Related ED Visits for the Total Population and Stratified by Preterm Birth Status
eTable 7. Weekly Association Between $PM_{2.5}$ Exposure and First All-Cause ED Stratified by Sex and Payment Type for Delivery
eFigure 1. Inclusion and Exclusion Criteria Flowchart
eFigure 2. 2014-2019 Timeseries of Average Weekly $PM_{2.5}$ Concentration
eFigure 3. Weekly Association Between $PM_{2.5}$ Exposure and First All-Cause ED Visits for the Total Population
eFigure 4. Weekly Association Between $PM_{2.5}$ Exposure and First Infection- and Respiratory-Related ED Visits Stratified by Preterm Birth Status
eFigure 5. Weekly Association Between $PM_{2.5}$ Exposure and First All-Cause ED Visits for Male and Female Infants
eFigure 6. Weekly Association Between $PM_{2.5}$ Exposure and First All-Cause ED Visits Stratified by Payment Type for Delivery

This supplemental material has been provided by the authors to give readers additional information about their work.

© 2023 Teyton A et al. *JAMA Network Open.*

eTable 1. Descriptive Statistics Before and After Excluding Infants With More Than 20 Weeks Between Birth and Discharge Dates

Descriptive statistics regarding weeks between birth date and discharge date for the total population and by preterm birth status. Provided before and after excluding infants with more than 20 weeks between birth date and discharge date.

Prior to excluding infants with more than 20 weeks between birth date and discharge date								
**Population**	**N**	**Mean**	**SD[a]**	**Minimum**	**1st Quartile**	**Median**	**3rd Quartile**	**Maximum**
Total Population	1,983,957	1.42	0.96	1	1	1	2	48
Term Only	1,841,648	1.32	0.56	1	1	1	2	44
Preterm Only	142,309	2.60	2.71	1	1	2	3	48
After excluding infants with more than 20 weeks between birth date and discharge date								
**Population**	**N**	**Mean**	**SD[a]**	**Minimum**	**1st Quartile**	**Median**	**3rd Quartile**	**Maximum**
Total Population	1,983,700	1.41	0.92	1	1	1	2	20
Term Only	1,841,619	1.32	0.55	1	1	1	2	20
Preterm Only	142,081	2.56	2.56	1	1	2	3	20

[a]SD: Standard Deviation

© 2023 Teyton A et al. *JAMA Network Open.*

eTable 2. Differential Exposure and Outcome by Complete Case Analysis and Total Population Including Individuals With Missing Covariates

Differences between the total population using a complete case analysis (n=1,983,700) and the total population of infants born in 2014-2018 with a California zip code including those with missing values for covariates (n=2,165,866).

	**Total**		**Average $PM_{2.5}$ Concentration (Standard Deviation)**		**Total with an ED Visit (%)**	
	Complete Case Analysis n=1,983,700	Full population including missing n=2,165,866	Complete Case Analysis	Full population including missing	Complete Case Analysis	Full population including missing
**Delivery Payment Type**						
Private Insurance	971,056	1,061,085	10.17 (2.53)	10.13 (2.53)	211,206 (21.75)	231,990 (21.84)
Medi-Cal	882,170	959,262	10.93 (3.19)	10.88 (3.18)	354,111 (40.14)	385,321 (40.10)
Self-Pay	68,426	73,868	11.35 (2.03)	11.31 (2.05)	7,065 (10.33)	7,744 (10.47)
Other/ Unknown	62,048	66,806	10.10 (2.56)	10.07 (2.53)	18,506 (29.83)	19,929 (29.79)
Missing	0	1,575	N/A	10.58 (2.84)	N/A	526 (33.25)
**Education**						
Less than high school	300,204	312,029	10.88 (3.18)	10.86 (3.18)	123,913 (41.28)	129,174 (41.32)
Greater than or equal to high school	1,683,496	1,748,387	10.49 (2.80)	10.47 (2.79)	466,975 (27.74)	488,031 (27.87)
Missing	0	102,180	N/A	10.00 (2.68)	N/A	28,305 (27.66)
**Parity**						
Nulliparous	747,383	815,956	10.47 (2.80)	10.42 (2.79)	219,327 (29.35)	239,694 (29.33)
Multiparous	1,246,317	1,345,232	10.60 (2.90)	10.55 (2.89)	371,561 (30.05)	405,420 (30.09)
Missing	0	1,408	N/A	11.13 (3.33)	N/A	396 (28.01)
**Discharge Date**						
Has discharge date	1,983,700	2,083,943	10.55 (2.86)	10.52 (2.85)	590,888 (29.79)	620,798 (29.74)
Missing	0	78,653	N/A	9.97 (2.76)	N/A	24,712 (31.39)

© 2023 Teyton A et al. *JAMA Network Open.*

eTable 3. Frequency of First Specific-Cause ED Visit for Total Population and Stratified by Preterm Birth Status

Classification for first specific-cause ED visit categories using ICD-9 and ICD-10 code ranges. First specific-cause ED visit frequencies and percentages are provided for all infants (n=590,888), term infants (n= 547,405), and preterm infants (n= 43,483) with an ED visit during their first year of life.

**Specific Cause Category**	**ICD-9 Code Range**	**ICD-10 Code Range**	**Frequency (%) for Total Population**	**Frequency (%) for Term Infants**	**Frequency (%) for Preterm Infants**
Symptoms, Signs, And Ill-Defined Conditions	780-799	R00-R99	159,775 (27.04%)	147,525 (26.95%)	12,250 (28.17%)
Diseases Of the Respiratory System[a]	460-519	J00-J99	154,780 (26.19%)	142,901 (26.11%)	11,879 (27.32%)
Certain Conditions Originating in the Perinatal Period	760-779	P00-P96	56,370 (9.54%)	52,717 (9.63%)	3,653 (8.40%)
Injury, Poisoning, and Certain Other Consequences of External Causes	800-999	S00-T88	47,569 (8.05%)	44,532 (8.14%)	3,037 (6.98%)
Infectious and Parasitic Diseases[b]	001-139	A00-B99	37,461 (6.34%)	34,750 (6.35%)	2,711 (6.23%)
Diseases Of the Digestive System	520-579	K00-K95	28,260 (4.78%)	25,557 (4.67%)	2,703 (6.22%)
Factors influencing health status and contact with health services	N/A	Z00-Z99	23,256 (3.94%)	21,497 (3.93%)	1,759 (4.05%)
Diseases Of the Skin and Subcutaneous Tissue	680-709	L00-L99	20,445 (3.46%)	19,222 (3.51%)	1,223 (2.81%)
Diseases of the Ear and Mastoid Process	N/A	H60-H95	14,324 (2.42%)	13,429 (2.45%)	895 (2.06%)
External causes of morbidity and mortality	N/A	V00-Y99	12,973 (2.20%)	12,088 (2.21%)	885 (2.04%)
Diseases Of the Genitourinary System	580-629	N00-N99	11,466 (1.94%)	10,708 (1.96%)	758 (1.74%)
Diseases Of the Nervous System and Sense Organs	320-389	G00-G99	9,761 (1.65%)	9,121 (1.67%)	640 (1.47%)
Diseases of the Eye and Adnexa	N/A	H00-H59	6,314 (1.07%)	5,882 (1.07%)	432 (0.99%)
Congenital Malformations, Deformations, and Chromosomal Abnormalities	740-759	Q00-Q99	2,364 (0.40%)	2,171 (0.40%)	193 (0.44%)
Endocrine, Nutritional and Metabolic Diseases, And Immunity Disorders	240-279	E00-E89	2,177 (0.37%)	2,012 (0.37%)	165 (0.38%)
Diseases Of the Musculoskeletal System and Connective Tissue	710-739	M00-M99	1,517 (0.26%)	1,422 (0.26%)	95 (0.22%)
Diseases Of the Circulatory System	390-459	I00-I99	929 (0.16%)	839 (0.15%)	90 (0.21%)
Diseases Of the Blood and Blood-Forming Organs	280-289	D50-D89	446 (0.08%)	398 (0.07%)	48 (0.11%)
Neoplasms	140-239	C00-D49	454 (0.08%)	408 (0.07%)	46 (0.11%)
Mental & Behavioral Disorders	290-319	F01-F99	243 (0.04%)	222 (0.04%)	21 (0.05%)
Complications Of Pregnancy, Childbirth, And the Puerperium	630-679	O00-O9A	4 (0.00%)	4 (0.00%)	0 (0.00%)

[a] Infants included in sub-analysis assessing the relationship between $PM_{2.5}$ exposure and first respiratory-related ED visit.
[b] Infants included in sub-analysis assessing the relationship between $PM_{2.5}$ exposure and first infection-related ED visit.

© 2023 Teyton A et al. *JAMA Network Open.*

eTable 4. Results from Cochran Q Test

**Variable**	**Q**	***P* value**
Preterm birth status	1.16	.28
Infection-related visit	1.02	.31
Respiratory-related visit	4.35	.04
Sex	0.25	.61
Delivery payment type	21.09	<.001

© 2023 Teyton A et al. *JAMA Network Open.*

eTable 5. Weekly Association Between $PM_{2.5}$ Exposure and First All-Cause ED Visits for the Total Population and Stratified by Preterm Birth Status

Adjusted odds ratios (AOR) and 95% confidence intervals (CI) for models that provide the weekly relationship between $PM_{2.5}$ exposure[a] and all-cause ED visits for the total population and stratified by preterm birth status.

	Total Population		Preterm Only		Term Only	
**Week**	**AOR**	**95% CI**	**AOR**	**95% CI**	**AOR**	**95% CI**
Week 1	1.027	1.012, 1.042	1.026	0.949, 1.111	1.027	1.012, 1.043
Week 2	1.027	1.012, 1.042	1.027	0.987, 1.068	1.010	0.999, 1.020
Week 3	1.011	1.001, 1.021	1.022	0.975, 1.070	1.021	1.009, 1.033
Week 4	1.011	1.001, 1.021	1.058	1.012, 1.106	1.035	1.022, 1.048
Week 5	1.021	1.010, 1.033	1.048	1.002, 1.096	1.043	1.030, 1.056
Week 6	1.021	1.010, 1.033	1.046	1.003, 1.091	1.048	1.035, 1.062
Week 7	1.036	1.024, 1.049	1.041	0.995, 1.089	1.029	1.014, 1.043
Week 8	1.036	1.024, 1.049	1.027	0.979, 1.078	1.039	1.024, 1.054
Week 9	1.043	1.031, 1.056	1.000	0.949, 1.053	1.049	1.035, 1.064
Week 10	1.043	1.031, 1.056	1.029	0.984, 1.077	1.024	1.008, 1.040
Week 11	1.048	1.036, 1.061	1.025	0.972, 1.081	1.036	1.019, 1.053
Week 12	1.048	1.036, 1.061	1.051	1.000, 1.104	1.032	1.014, 1.049
Week 13	1.030	1.016, 1.044	1.023	0.969, 1.080	1.033	1.016, 1.050
Week 14	1.030	1.016, 1.044	1.057	1.002, 1.114	1.036	1.019, 1.054
Week 15	1.038	1.024, 1.053	1.034	0.977, 1.096	1.037	1.020, 1.055
Week 16	1.038	1.024, 1.053	0.999	0.937, 1.065	1.048	1.031, 1.065
Week 17	1.046	1.032, 1.060	1.004	0.943, 1.069	1.048	1.031, 1.065
Week 18	1.046	1.032, 1.060	1.066	1.011, 1.123	1.046	1.029, 1.064
Week 19	1.025	1.009, 1.040	1.034	0.976, 1.094	1.076	1.060, 1.093
Week 20	1.025	1.009, 1.040	1.077	1.022, 1.135	1.037	1.019, 1.056
Week 21	1.035	1.019, 1.051	1.053	0.997, 1.112	1.062	1.044, 1.080
Week 22	1.035	1.019, 1.051	1.064	1.006, 1.125	1.042	1.024, 1.061
Week 23	1.034	1.017, 1.050	1.052	0.988, 1.120	1.042	1.025, 1.060
Week 24	1.034	1.017, 1.050	1.024	0.963, 1.089	1.031	1.013, 1.050
Week 25	1.032	1.016, 1.049	1.008	0.946, 1.073	1.021	1.003, 1.039
Week 26	1.032	1.016, 1.049	0.976	0.908, 1.050	1.031	1.013, 1.049
Week 27	1.038	1.022, 1.055	1.024	0.959, 1.092	1.030	1.012, 1.048
Week 28	1.038	1.022, 1.055	1.048	0.994, 1.104	1.047	1.030, 1.064
Week 29	1.037	1.021, 1.054	1.042	0.983, 1.104	1.036	1.019, 1.053
Week 30	1.037	1.021, 1.054	1.010	0.944, 1.082	1.025	1.007, 1.044
Week 31	1.045	1.028, 1.061	1.070	1.014, 1.129	1.028	1.009, 1.047
Week 32	1.045	1.028, 1.061	1.035	0.971, 1.103	1.046	1.027, 1.065
Week 33	1.045	1.028, 1.061	0.995	0.923, 1.072	1.045	1.027, 1.064
Week 34	1.045	1.028, 1.061	1.029	0.960, 1.102	1.048	1.029, 1.068
Week 35	1.048	1.031, 1.065	1.062	1.001, 1.128	1.036	1.017, 1.056
Week 36	1.048	1.031, 1.065	1.016	0.951, 1.086	1.015	0.996, 1.034
Week 37	1.073	1.057, 1.089	1.005	0.939, 1.076	1.023	1.005, 1.042
Week 38	1.073	1.057, 1.089	1.004	0.935, 1.077	1.045	1.028, 1.062
Week 39	1.041	1.023, 1.059	1.071	1.008, 1.138	1.037	1.020, 1.055
Week 40	1.041	1.023, 1.059	0.978	0.899, 1.064	1.025	1.006, 1.044
Week 41	1.061	1.044, 1.078	0.989	0.916, 1.067	1.032	1.014, 1.051
Week 42	1.061	1.044, 1.078	1.047	0.988, 1.110	1.030	1.011, 1.049
Week 43	1.044	1.027, 1.062	1.062	1.003, 1.124	1.021	1.001, 1.041
Week 44	1.044	1.027, 1.062	0.989	0.912, 1.072	1.024	1.004, 1.045
Week 45	1.043	1.026, 1.060	1.002	0.926, 1.085	1.024	1.004, 1.045
Week 46	1.043	1.026, 1.060	1.091	1.024, 1.163	1.031	1.010, 1.053
Week 47	1.031	1.013, 1.048	1.056	0.980, 1.137	1.031	1.008, 1.055

© 2023 Teyton A et al. *JAMA Network Open.*

	Total Population		Preterm Only		Term Only	
**Week**	**AOR**	**95% CI**	**AOR**	**95% CI**	**AOR**	**95% CI**
Week 48	1.031	1.013, 1.048	1.005	0.922, 1.096	1.039	1.018, 1.061
Week 49	1.020	1.002, 1.037	1.032	0.965, 1.103	1.033	1.013, 1.053
Week 50	1.020	1.002, 1.037	1.054	0.989, 1.123	1.023	1.002, 1.044
Week 51	1.027	1.010, 1.045	1.050	0.986, 1.118	1.016	0.995, 1.037
Week 52	1.027	1.010, 1.045	1.012	0.936, 1.094	1.027	1.007, 1.048

[a]$PM_{2.5}$ exposure is included in increments of 5 μg/m^3.

© 2023 Teyton A et al. *JAMA Network Open.*

eTable 6. Weekly Association Between $PM_{2.5}$ Exposure and First Infection- and Respiratory-Related ED Visits for the Total Population and Stratified by Preterm Birth Status

Adjusted odds ratios (AOR) and 95% confidence intervals (CIs) for the models that provide the weekly relationship between $PM_{2.5}$ exposure[a] and specific-cause ED visits, including infections and respiratory diseases.

	Infection-Related ED Visit						Respiratory-Related ED Visit					
	Total Population		Preterm Only		Term Only		Total Population		Preterm Only		Term Only	
Week	AOR	95% CI	AOR	95% CI	AOR	95% CI	AOR	95% CI	AOR	95% CI	AOR	95% CI
Week 1	0.819	0.456, 1.471	1.038	0.017, 61.973	0.759	0.401, 1.436	1.186	1.070, 1.313	1.384	0.994, 1.926	1.180	1.041, 1.338
Week 2	1.032	0.906, 1.176	0.792	0.345, 1.818	1.041	0.914, 1.186	1.063	1.007, 1.122	0.979	0.687, 1.394	1.066	1.010, 1.126
Week 3	1.070	0.987, 1.161	0.992	0.659, 1.494	1.073	0.989, 1.165	1.094	1.058, 1.132	1.052	0.889, 1.245	1.096	1.059, 1.135
Week 4	1.104	1.043, 1.170	0.996	0.720, 1.377	1.110	1.047, 1.176	1.080	1.047, 1.114	1.134	1.027, 1.254	1.076	1.041, 1.111
Week 5	1.061	1.001, 1.124	0.935	0.698, 1.255	1.068	1.008, 1.132	1.078	1.050, 1.106	1.067	0.952, 1.195	1.078	1.050, 1.108
Week 6	1.050	0.994, 1.109	1.010	0.818, 1.246	1.053	0.995, 1.115	1.086	1.063, 1.110	1.062	0.975, 1.156	1.088	1.064, 1.113
Week 7	1.060	1.007, 1.116	1.109	0.961, 1.280	1.056	0.999, 1.115	1.075	1.050, 1.100	1.132	1.062, 1.206	1.068	1.042, 1.095
Week 8	1.071	1.014, 1.130	1.061	0.860, 1.308	1.072	1.013, 1.133	1.078	1.054, 1.101	1.081	1.005, 1.163	1.077	1.053, 1.102
Week 9	1.066	1.013, 1.121	1.157	1.019, 1.314	1.053	0.997, 1.113	1.071	1.048, 1.096	0.990	0.893, 1.098	1.077	1.053, 1.102
Week 10	1.030	0.968, 1.096	0.885	0.635, 1.234	1.040	0.976, 1.108	1.035	1.008, 1.062	1.023	0.935, 1.120	1.035	1.007, 1.064
Week 11	0.974	0.903, 1.051	0.878	0.609, 1.266	0.980	0.907, 1.058	1.072	1.046, 1.098	1.056	0.966, 1.155	1.073	1.046, 1.101
Week 12	0.981	0.909, 1.059	1.116	0.939, 1.326	0.963	0.886, 1.047	1.051	1.025, 1.077	1.052	0.960, 1.154	1.050	1.023, 1.078
Week 13	1.006	0.938, 1.079	0.995	0.753, 1.315	1.006	0.936, 1.081	1.064	1.040, 1.088	1.055	0.966, 1.151	1.064	1.039, 1.090
Week 14	1.011	0.939, 1.088	1.069	0.857, 1.334	1.005	0.929, 1.087	1.067	1.041, 1.092	1.062	0.957, 1.177	1.067	1.041, 1.093
Week 15	1.008	0.940, 1.081	0.943	0.705, 1.261	1.012	0.942, 1.088	1.074	1.050, 1.098	1.072	0.991, 1.160	1.074	1.049, 1.099
Week 16	1.041	0.978, 1.108	0.947	0.712, 1.259	1.049	0.983, 1.119	1.070	1.046, 1.094	1.020	0.930, 1.120	1.073	1.049, 1.098
Week 17	1.071	1.010, 1.135	1.061	0.861, 1.307	1.072	1.008, 1.139	1.061	1.038, 1.085	1.005	0.915, 1.105	1.066	1.041, 1.091
Week 18	1.086	1.030, 1.146	1.237	0.964, 1.586	1.081	1.023, 1.143	1.060	1.036, 1.084	1.048	0.961, 1.144	1.061	1.036, 1.086
Week 19	1.108	1.053, 1.166	1.001	0.745, 1.344	1.112	1.056, 1.171	1.086	1.063, 1.110	1.062	0.986, 1.144	1.088	1.064, 1.113
Week 20	1.045	0.983, 1.111	1.025	0.836, 1.256	1.047	0.982, 1.117	1.063	1.038, 1.089	1.100	1.016, 1.191	1.060	1.034, 1.087
Week 21	1.055	0.993, 1.122	1.029	0.807, 1.311	1.057	0.993, 1.126	1.076	1.051, 1.100	1.047	0.956, 1.145	1.078	1.053, 1.104
Week 22	1.036	0.972, 1.103	1.054	0.862, 1.289	1.034	0.967, 1.104	1.060	1.034, 1.087	1.032	0.942, 1.132	1.063	1.036, 1.090
Week 23	1.025	0.966, 1.087	0.933	0.680, 1.279	1.030	0.970, 1.093	1.073	1.049, 1.097	1.097	1.013, 1.188	1.071	1.046, 1.096
Week 24	0.931	0.859, 1.009	0.973	0.769, 1.231	0.926	0.850, 1.008	1.050	1.023, 1.077	1.098	1.025, 1.175	1.043	1.015, 1.072
Week 25	1.009	0.947, 1.074	1.118	0.929, 1.344	0.999	0.935, 1.068	1.041	1.016, 1.067	1.039	0.948, 1.138	1.041	1.015, 1.068
Week 26	1.058	1.002, 1.116	0.890	0.669, 1.184	1.068	1.012, 1.127	1.042	1.017, 1.068	0.971	0.869, 1.084	1.047	1.021, 1.073
Week 27	0.958	0.892, 1.030	0.919	0.656, 1.286	0.961	0.893, 1.034	1.052	1.026, 1.078	1.064	0.977, 1.158	1.050	1.024, 1.078
Week 28	1.035	0.977, 1.096	1.100	0.915, 1.323	1.030	0.969, 1.094	1.073	1.050, 1.096	1.088	1.020, 1.160	1.071	1.046, 1.096
Week 29	1.055	0.995, 1.118	1.162	1.017, 1.327	1.042	0.979, 1.110	1.057	1.033, 1.081	1.058	0.974, 1.150	1.057	1.032, 1.082
Week 30	1.030	0.966, 1.098	0.801	0.588, 1.093	1.044	0.980, 1.112	1.069	1.044, 1.094	1.081	0.987, 1.183	1.068	1.043, 1.094
Week 31	1.009	0.947, 1.076	0.995	0.767, 1.291	1.011	0.946, 1.08	1.049	1.021, 1.078	1.082	0.996, 1.174	1.045	1.016, 1.076

© 2023 Teyton A et al. *JAMA Network Open.*

	Infection-Related ED Visit						Respiratory-Related ED Visit					
	Total Population		Preterm Only		Term Only		Total Population		Preterm Only		Term Only	
**Week**	**AOR**	**95% CI**	**AOR**	**95% CI**	**AOR**	**95% CI**	**AOR**	**95% CI**	**AOR**	**95% CI**	**AOR**	**95% CI**
Week 32	0.992	0.924, 1.064	0.873	0.660, 1.153	1.003	0.932, 1.079	1.066	1.039, 1.093	1.076	0.981, 1.180	1.065	1.037, 1.093
Week 33	0.984	0.918, 1.054	0.923	0.663, 1.283	0.987	0.920, 1.059	1.064	1.037, 1.092	1.045	0.949, 1.151	1.066	1.038, 1.095
Week 34	1.058	0.996, 1.123	0.824	0.572, 1.187	1.070	1.008, 1.136	1.045	1.016, 1.075	1.084	0.993, 1.182	1.041	1.010, 1.072
Week 35	0.978	0.908, 1.054	0.878	0.642, 1.202	0.985	0.913, 1.063	1.064	1.037, 1.092	1.067	0.977, 1.166	1.064	1.036, 1.093
Week 36	1.000	0.942, 1.062	0.631	0.408, 0.978	1.013	0.956, 1.074	1.030	1.001, 1.061	1.013	0.919, 1.118	1.032	1.001, 1.064
Week 37	0.983	0.920, 1.049	0.745	0.506, 1.097	0.994	0.932, 1.061	1.070	1.045, 1.095	1.038	0.939, 1.148	1.072	1.046, 1.098
Week 38	0.999	0.934, 1.068	0.906	0.669, 1.227	1.005	0.939, 1.076	1.072	1.047, 1.097	1.019	0.918, 1.131	1.076	1.050, 1.102
Week 39	1.036	0.982, 1.093	1.195	1.042, 1.371	1.021	0.963, 1.083	1.062	1.035, 1.089	1.082	0.977, 1.199	1.060	1.033, 1.088
Week 40	0.980	0.915, 1.049	0.843	0.604, 1.175	0.988	0.922, 1.059	1.042	1.012, 1.072	1.026	0.911, 1.154	1.043	1.012, 1.074
Week 41	0.969	0.901, 1.041	0.952	0.739, 1.227	0.970	0.899, 1.046	1.061	1.034, 1.090	1.081	0.962, 1.215	1.061	1.032, 1.090
Week 42	1.018	0.959, 1.080	0.896	0.633, 1.268	1.023	0.964, 1.086	1.051	1.023, 1.081	1.050	0.967, 1.141	1.051	1.021, 1.082
Week 43	0.978	0.912, 1.050	0.976	0.705, 1.353	0.979	0.911, 1.052	1.047	1.017, 1.077	1.044	0.949, 1.150	1.047	1.016, 1.079
Week 44	1.000	0.933, 1.072	0.583	0.362, 0.939	1.019	0.953, 1.090	1.065	1.036, 1.094	1.067	0.955, 1.192	1.065	1.035, 1.095
Week 45	1.013	0.951, 1.078	0.760	0.485, 1.190	1.020	0.960, 1.085	1.035	1.003, 1.069	1.095	0.992, 1.208	1.030	0.996, 1.065
Week 46	1.049	0.988, 1.114	1.311	1.094, 1.571	1.034	0.969, 1.105	1.044	1.010, 1.081	1.064	0.934, 1.212	1.043	1.007, 1.081
Week 47	1.044	0.974, 1.118	1.210	1.069, 1.371	1.015	0.937, 1.099	1.037	1.000, 1.076	1.052	0.912, 1.213	1.036	0.997, 1.076
Week 48	1.039	0.972, 1.111	1.038	0.807, 1.333	1.039	0.970, 1.114	1.045	1.009, 1.083	1.048	0.918, 1.196	1.046	1.008, 1.084
Week 49	0.980	0.901, 1.064	1.021	0.781, 1.335	0.975	0.894, 1.065	1.063	1.033, 1.095	1.062	0.952, 1.185	1.063	1.032, 1.096
Week 50	1.052	0.998, 1.109	1.085	0.790, 1.492	1.052	0.997, 1.110	1.033	0.999, 1.067	1.082	0.992, 1.179	1.026	0.990, 1.063
Week 51	0.981	0.908, 1.060	1.062	0.818, 1.380	0.974	0.898, 1.057	1.025	0.991, 1.060	1.093	0.982, 1.217	1.019	0.983, 1.056
Week 52	1.004	0.938, 1.074	1.002	0.784, 1.282	1.004	0.935, 1.077	1.053	1.020, 1.088	1.085	0.988, 1.192	1.050	1.014, 1.087

[a]$PM_{2.5}$ exposure is included in increments of 5 μg/m^3

© 2023 Teyton A et al. *JAMA Network Open.*

eTable 7. Weekly Association Between $PM_{2.5}$ Exposure and First All-Cause ED Stratified by Sex and Payment Type for Delivery

Adjusted odds ratios (AOR) and 95% confidence intervals (CIs) for the models that provide the week-by-week relationship between $PM_{2.5}$ exposure[a] and all-cause ED visits stratified by sex and by payment type for the delivery.

	Sex				Payment Type for Delivery							
	Male		Female		Private		Medi-Cal		Self-Paid		Other/ Unknown	
Week	AOR	95% CI	AOR	95% CI	AOR	95% CI	AOR	95% CI	AOR	95% CI	AOR	95% CI
Week 1	1.014	0.993, 1.036	1.040	1.019, 1.062	1.011	0.986, 1.037	1.032	1.013, 1.052	1.216	1.077, 1.373	1.076	0.978, 1.184
Week 2	1.006	0.992, 1.020	1.016	1.001, 1.030	1.010	0.993, 1.028	1.013	1.001, 1.025	1.012	0.906, 1.131	1.028	0.965, 1.095
Week 3	1.018	1.002, 1.034	1.025	1.008, 1.042	1.011	0.990, 1.033	1.026	1.013, 1.040	0.879	0.742, 1.042	1.046	0.972, 1.127
Week 4	1.040	1.023, 1.057	1.032	1.014, 1.051	1.032	1.009, 1.056	1.038	1.023, 1.053	1.060	0.904, 1.244	1.058	0.979, 1.143
Week 5	1.045	1.028, 1.063	1.041	1.022, 1.060	1.040	1.016, 1.064	1.044	1.029, 1.060	1.098	0.942, 1.281	1.040	0.961, 1.126
Week 6	1.051	1.034, 1.068	1.045	1.026, 1.064	1.042	1.018, 1.066	1.050	1.035, 1.065	1.079	0.932, 1.250	1.084	0.999, 1.176
Week 7	1.033	1.014, 1.053	1.027	1.007, 1.047	1.034	1.010, 1.060	1.027	1.010, 1.044	1.029	0.869, 1.218	1.051	0.961, 1.150
Week 8	1.038	1.018, 1.058	1.039	1.018, 1.060	1.047	1.022, 1.072	1.034	1.016, 1.052	1.041	0.894, 1.212	1.078	0.982, 1.183
Week 9	1.047	1.028, 1.067	1.044	1.024, 1.064	1.047	1.022, 1.072	1.045	1.027, 1.063	1.065	0.898, 1.263	1.072	0.991, 1.159
Week 10	1.025	1.004, 1.047	1.024	1.002, 1.047	1.016	0.988, 1.045	1.026	1.008, 1.045	1.158	1.002, 1.338	1.042	0.942, 1.153
Week 11	1.041	1.020, 1.063	1.028	1.005, 1.052	1.030	1.002, 1.059	1.038	1.018, 1.058	1.111	0.918, 1.344	0.973	0.855, 1.108
Week 12	1.040	1.018, 1.063	1.026	1.002, 1.051	1.041	1.013, 1.071	1.029	1.009, 1.049	1.113	0.942, 1.314	1.034	0.926, 1.154
Week 13	1.020	0.997, 1.043	1.045	1.023, 1.068	1.024	0.995, 1.055	1.038	1.018, 1.058	1.049	0.861, 1.279	0.946	0.823, 1.088
Week 14	1.042	1.019, 1.065	1.034	1.010, 1.058	1.036	1.007, 1.066	1.038	1.018, 1.059	1.218	1.005, 1.476	1.042	0.933, 1.163
Week 15	1.039	1.017, 1.063	1.035	1.011, 1.060	1.052	1.023, 1.082	1.030	1.009, 1.050	1.169	0.971, 1.408	1.073	0.977, 1.179
Week 16	1.051	1.029, 1.074	1.038	1.014, 1.062	1.060	1.033, 1.088	1.038	1.017, 1.058	1.112	0.904, 1.367	0.975	0.856, 1.110
Week 17	1.044	1.022, 1.068	1.045	1.021, 1.070	1.060	1.032, 1.088	1.036	1.015, 1.057	1.197	0.954, 1.501	1.043	0.933, 1.165
Week 18	1.044	1.021, 1.068	1.052	1.028, 1.076	1.058	1.031, 1.087	1.037	1.016, 1.059	1.217	0.945, 1.566	1.134	1.051, 1.224
Week 19	1.080	1.059, 1.101	1.064	1.040, 1.088	1.095	1.070, 1.121	1.059	1.039, 1.080	1.117	0.939, 1.330	1.054	0.936, 1.187
Week 20	1.035	1.011, 1.060	1.047	1.022, 1.072	1.054	1.025, 1.084	1.033	1.011, 1.055	1.239	1.064, 1.443	1.024	0.887, 1.182
Week 21	1.074	1.051, 1.097	1.046	1.021, 1.072	1.079	1.050, 1.109	1.051	1.030, 1.073	1.219	1.074, 1.382	1.025	0.908, 1.159
Week 22	1.041	1.017, 1.066	1.048	1.023, 1.073	1.064	1.034, 1.094	1.035	1.013, 1.058	1.198	0.994, 1.444	1.021	0.897, 1.162
Week 23	1.050	1.028, 1.073	1.034	1.009, 1.060	1.056	1.027, 1.087	1.035	1.015, 1.056	1.257	1.062, 1.487	1.033	0.915, 1.165
Week 24	1.037	1.013, 1.062	1.023	0.997, 1.049	1.061	1.034, 1.089	1.011	0.988, 1.034	0.963	0.722, 1.284	1.051	0.916, 1.207
Week 25	1.007	0.982, 1.032	1.032	1.008, 1.056	1.028	0.998, 1.058	1.015	0.993, 1.037	1.089	0.898, 1.321	1.056	0.960, 1.162
Week 26	1.031	1.008, 1.056	1.023	0.998, 1.048	1.034	1.005, 1.065	1.019	0.998, 1.042	1.168	0.982, 1.389	1.098	1.006, 1.199
Week 27	1.038	1.016, 1.062	1.018	0.993, 1.045	1.040	1.012, 1.068	1.022	1.000, 1.044	1.026	0.831, 1.268	1.062	0.936, 1.205
Week 28	1.040	1.017, 1.063	1.054	1.032, 1.077	1.042	1.014, 1.070	1.052	1.033, 1.073	0.839	0.628, 1.120	1.043	0.938, 1.159
Week 29	1.044	1.022, 1.066	1.028	1.003, 1.053	1.046	1.020, 1.073	1.027	1.006, 1.049	1.079	0.893, 1.303	1.095	1.003, 1.196
Week 30	1.032	1.008, 1.056	1.014	0.988, 1.041	1.031	1.002, 1.061	1.018	0.996, 1.041	1.031	0.819, 1.298	1.040	0.929, 1.165
Week 31	1.034	1.009, 1.059	1.030	1.004, 1.057	1.051	1.023, 1.079	1.017	0.994, 1.042	0.871	0.648, 1.169	1.108	1.005, 1.221
Week 32	1.049	1.025, 1.074	1.040	1.015, 1.067	1.061	1.034, 1.089	1.031	1.008, 1.056	0.939	0.714, 1.234	1.129	0.989, 1.289

© 2023 Teyton A et al. *JAMA Network Open.*

Week	Sex				Payment Type for Delivery							
	Male		Female		Private		Medi-Cal		Self-Paid		Other/Unknown	
	AOR	95% CI	AOR	95% CI	AOR	95% CI	AOR	95% CI	AOR	95% CI	AOR	95% CI
Week 33	1.043	1.018, 1.068	1.041	1.016, 1.068	1.044	1.014, 1.074	1.042	1.020, 1.066	0.984	0.753, 1.286	1.037	0.916, 1.175
Week 34	1.048	1.023, 1.074	1.046	1.019, 1.073	1.082	1.055, 1.109	1.017	0.992, 1.043	1.079	0.829, 1.405	1.084	0.968, 1.213
Week 35	1.047	1.022, 1.073	1.029	1.001, 1.057	1.062	1.034, 1.091	1.025	1.000, 1.051	1.080	0.904, 1.290	0.862	0.719, 1.033
Week 36	1.016	0.991, 1.041	1.014	0.988, 1.040	1.022	0.994, 1.052	1.007	0.984, 1.031	0.915	0.667, 1.253	1.074	0.985, 1.170
Week 37	1.021	0.998, 1.046	1.022	0.996, 1.049	1.020	0.992, 1.048	1.022	0.999, 1.045	1.175	1.001, 1.379	1.025	0.897, 1.172
Week 38	1.033	1.010, 1.056	1.053	1.030, 1.077	1.052	1.027, 1.077	1.035	1.013, 1.057	1.057	0.841, 1.329	1.017	0.870, 1.190
Week 39	1.041	1.019, 1.064	1.037	1.012, 1.063	1.051	1.025, 1.078	1.032	1.010, 1.055	1.147	1.014, 1.297	0.977	0.852, 1.120
Week 40	1.011	0.985, 1.038	1.033	1.007, 1.059	1.026	0.997, 1.056	1.020	0.996, 1.044	0.898	0.663, 1.215	1.051	0.944, 1.169
Week 41	1.031	1.007, 1.056	1.028	1.002, 1.055	1.035	1.007, 1.063	1.025	1.001, 1.049	1.093	0.928, 1.287	1.073	0.949, 1.213
Week 42	1.008	0.981, 1.036	1.053	1.029, 1.077	1.041	1.014, 1.069	1.024	1.000, 1.049	1.088	0.864, 1.371	1.010	0.869, 1.174
Week 43	1.012	0.985, 1.039	1.037	1.011, 1.063	1.029	1.000, 1.058	1.012	0.986, 1.038	1.140	1.023, 1.271	1.167	1.070, 1.272
Week 44	1.030	1.004, 1.057	1.013	0.983, 1.042	1.026	0.996, 1.057	1.012	0.986, 1.039	1.162	0.948, 1.426	1.117	1.013, 1.231
Week 45	1.019	0.991, 1.047	1.028	1.000, 1.056	1.035	1.005, 1.065	1.014	0.987, 1.042	0.974	0.709, 1.340	1.022	0.896, 1.165
Week 46	1.054	1.027, 1.081	1.014	0.983, 1.045	1.059	1.030, 1.088	1.014	0.986, 1.042	0.840	0.601, 1.174	1.087	0.966, 1.223
Week 47	1.046	1.017, 1.076	1.016	0.982, 1.050	1.047	1.014, 1.081	1.021	0.991, 1.052	1.193	1.020, 1.395	0.961	0.775, 1.191
Week 48	1.023	0.994, 1.054	1.050	1.022, 1.079	1.031	1.000, 1.064	1.039	1.011, 1.067	0.934	0.693, 1.259	1.093	0.986, 1.211
Week 49	1.042	1.016, 1.068	1.021	0.992, 1.051	1.052	1.025, 1.080	1.013	0.985, 1.042	1.225	1.064, 1.411	0.990	0.828, 1.184
Week 50	1.030	1.003, 1.057	1.020	0.991, 1.049	1.032	1.004, 1.061	1.020	0.993, 1.049	1.008	0.765, 1.328	0.941	0.786, 1.127
Week 51	1.016	0.988, 1.044	1.022	0.994, 1.051	1.023	0.994, 1.053	1.005	0.978, 1.034	1.018	0.755, 1.372	1.164	1.059, 1.279
Week 52	1.024	0.996, 1.052	1.028	1.000, 1.057	1.008	0.976, 1.041	1.033	1.007, 1.060	1.127	0.957, 1.327	1.098	1.001, 1.205

[a] $PM_{2.5}$ exposure is included in increments of 5 μg/m^3

© 2023 Teyton A et al. *JAMA Network Open.*

eFigure 1. Inclusion and Exclusion Criteria Flowchart

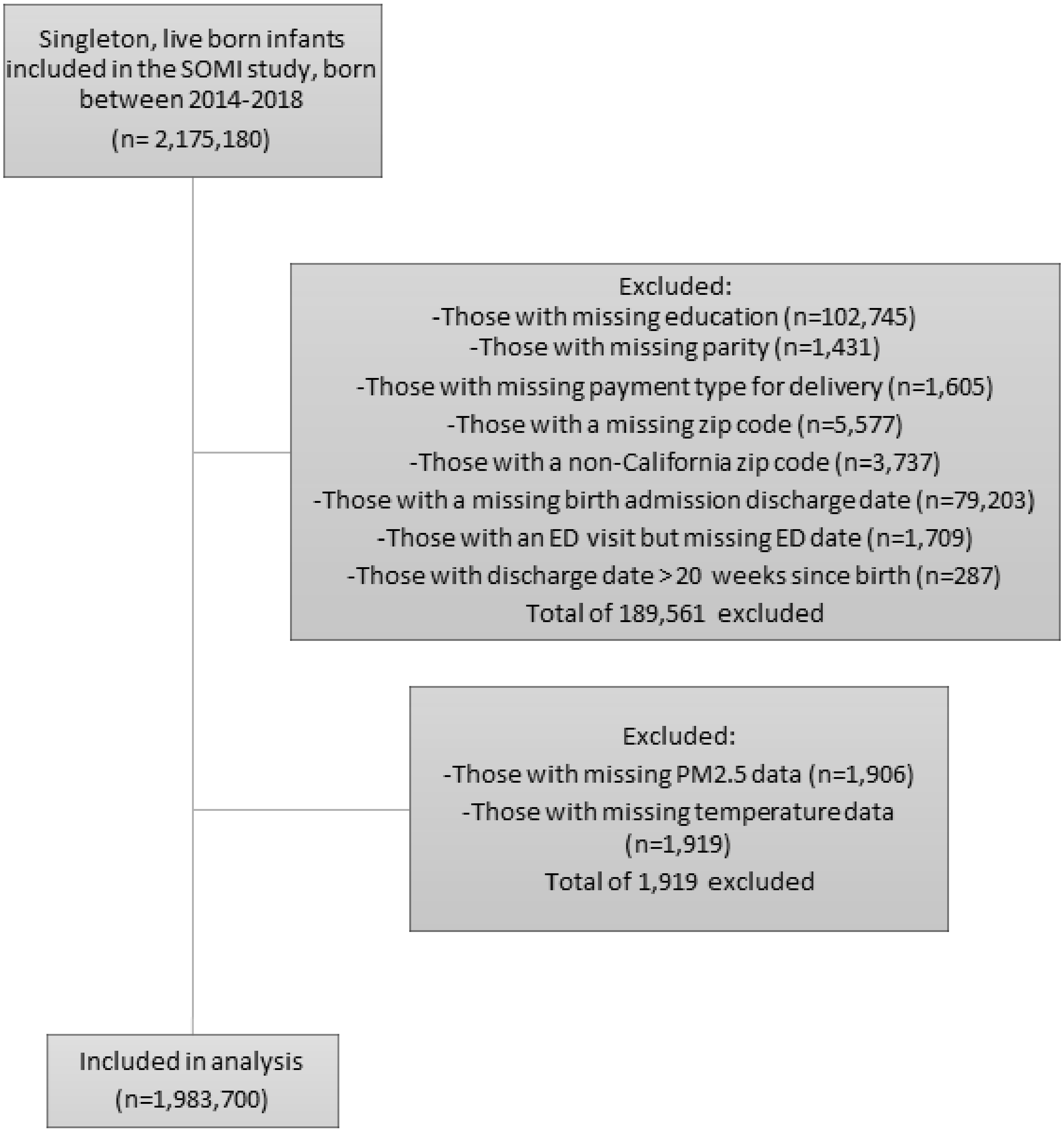

Flowchart providing inclusion and exclusion criteria for analytic study population (n=1,983,700). Note: There is overlap in missingness within each of the two exclusion boxes (e.g., an individual missing both parity and education values will be included in both the 1,431 and 102,745 counts), and the sum of those excluded from each box is provided. Those with missing covariates (education, parity, etc.) were removed prior to removing those with missing $PM_{2.5}$ and temperature data.

© 2023 Teyton A et al. *JAMA Network Open.*

eFigure 2. 2014-2019 Timeseries of Average Weekly $PM_{2.5}$ Concentration

Timeseries from 2014 through 2019 of the average weekly $PM_{2.5}$ concentration (micrograms per cubic meter) for infants during their time at risk.

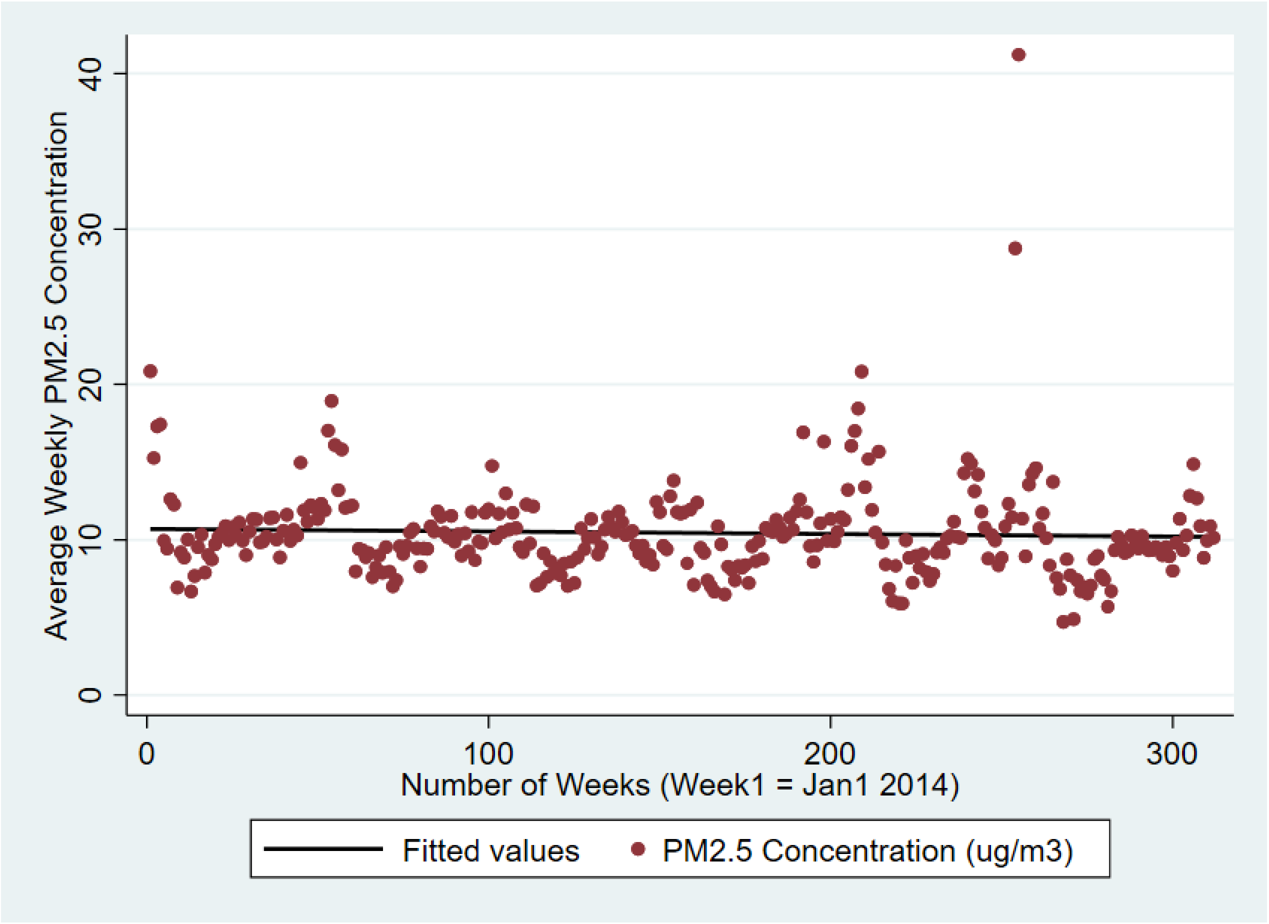

© 2023 Teyton A et al. *JAMA Network Open.*

eFigure 3. Weekly Association Between $PM_{2.5}$ Exposure and First All-Cause ED Visits for the Total Population

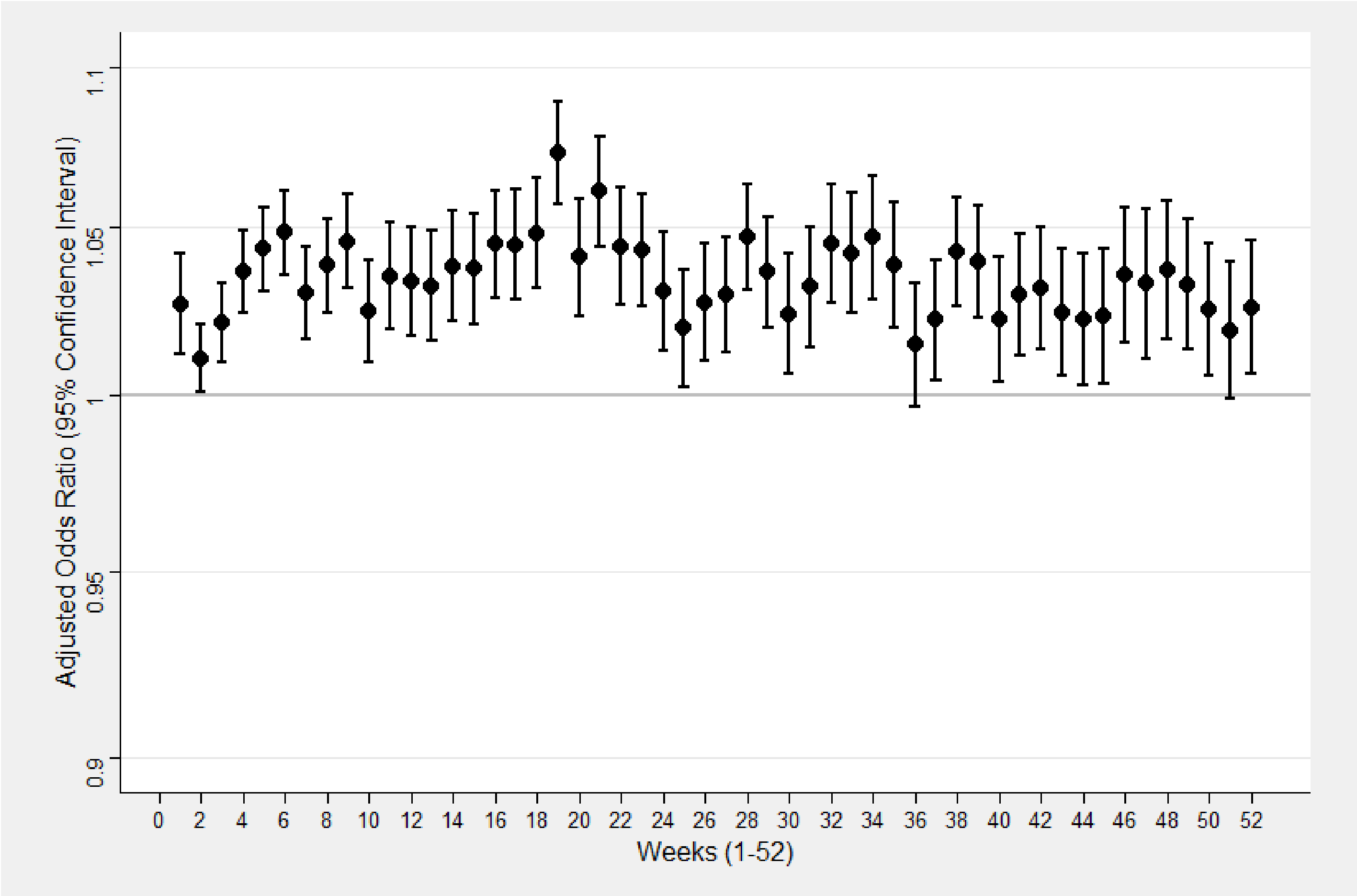

Models using a discrete time approach assessing the relationship between $PM_{2.5}$ exposure (increments of 5 $\mu g/m^3$) and time to all-cause ED visits during each week of the first year of life, where adjusted odds ratios and 95% confidence intervals are provided for the total population. Models were adjusted for payment type for delivery, parity, education, race and ethnicity, seasonality, and time-varying temperature. Y axis provided on the logarithmic scale.

© 2023 Teyton A et al. *JAMA Network Open.*

eFigure 4. Weekly Association Between $PM_{2.5}$ Exposure and First Infection- and Respiratory-Related ED Visits Stratified by Preterm Birth Status

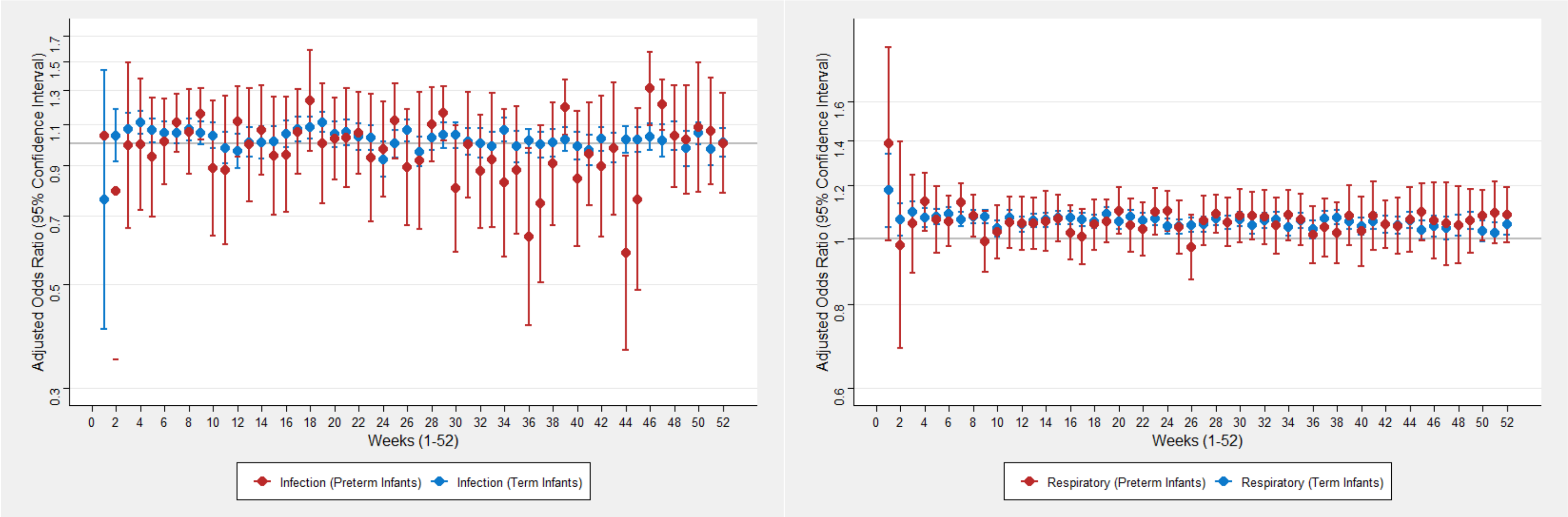

Models using a discrete time approach assessing the relationship between $PM_{2.5}$ exposure (increments of 5 $\mu g/m^3$) and time to infection- (left) and respiratory-related (right) ED visits during each week of the first year of life, where adjusted odds ratios and 95% confidence intervals are provided for preterm infants (red) and term infants (blue). In each model, specific causes of ED visits other than the one of interest were removed due to competing risks. These models were adjusted for payment type for delivery, parity, education, race and ethnicity, seasonality, and time-varying. Y axes provided on the logarithmic scale.
Note: The 95% confidence intervals for Weeks 1-2 are not shown for infection-related ED visits in preterm infants, given the wide upper limits. The tabular results are provided in eTable 5.

© 2023 Teyton A et al. *JAMA Network Open.*

eFigure 5. Weekly Association Between $PM_{2.5}$ Exposure and First All-Cause ED Visits for Male and Female Infants

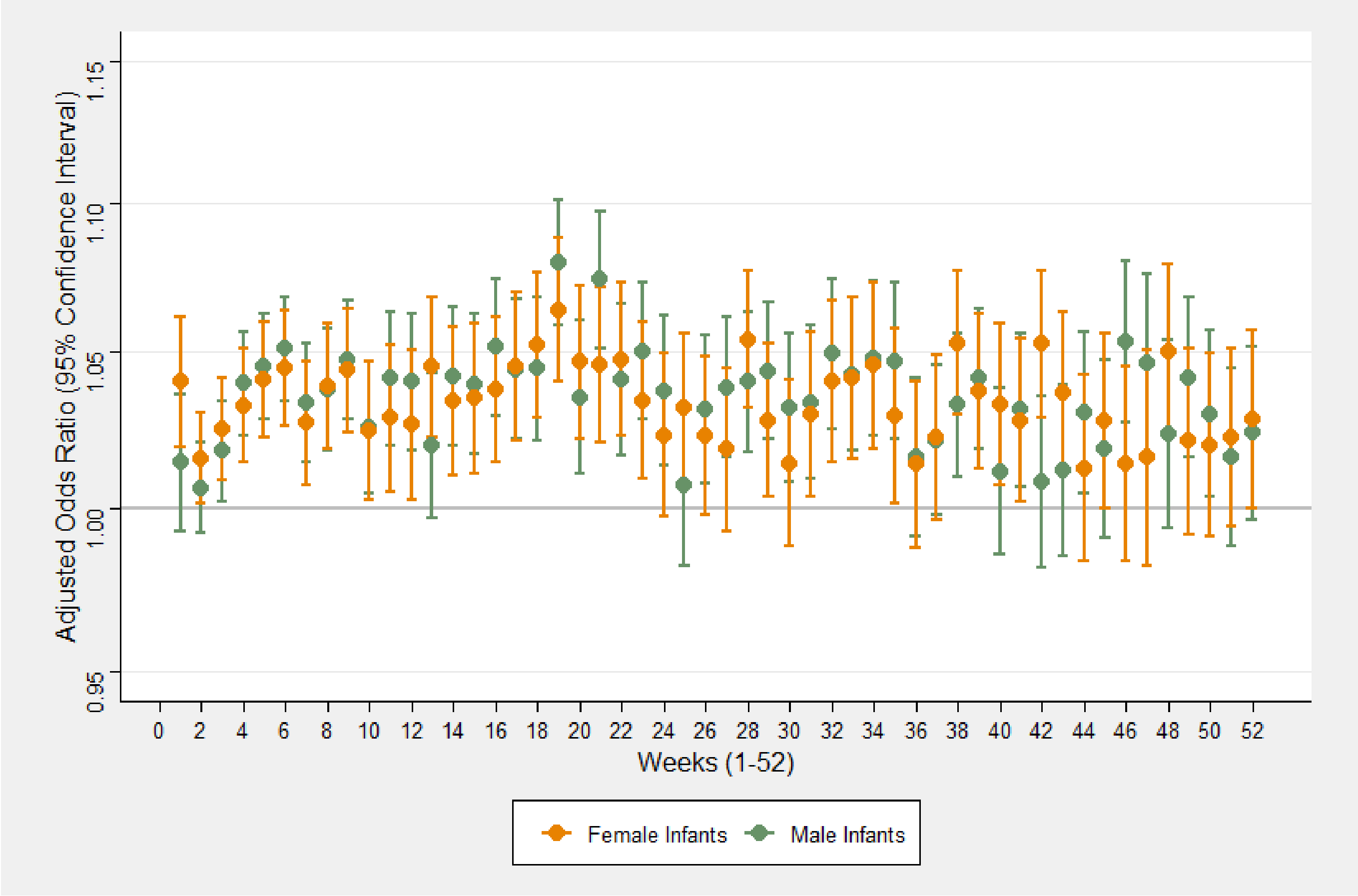

Models using a discrete time approach assessing the relationship between $PM_{2.5}$ exposure (increments of 5 μg/m^3) and time to all-cause ED visits during each week of the first year of life, where adjusted odds ratios and 95% confidence intervals are provided the total population stratified by sex (female in orange, male in green). These models were adjusted for payment type for delivery, parity, education, race and ethnicity, seasonality, and time-varying temperature. Y axis provided on the logarithmic scale.

© 2023 Teyton A et al. *JAMA Network Open.*

eFigure 6. Weekly Association Between $PM_{2.5}$ Exposure and First All-Cause ED Visits Stratified by Payment Type for Delivery

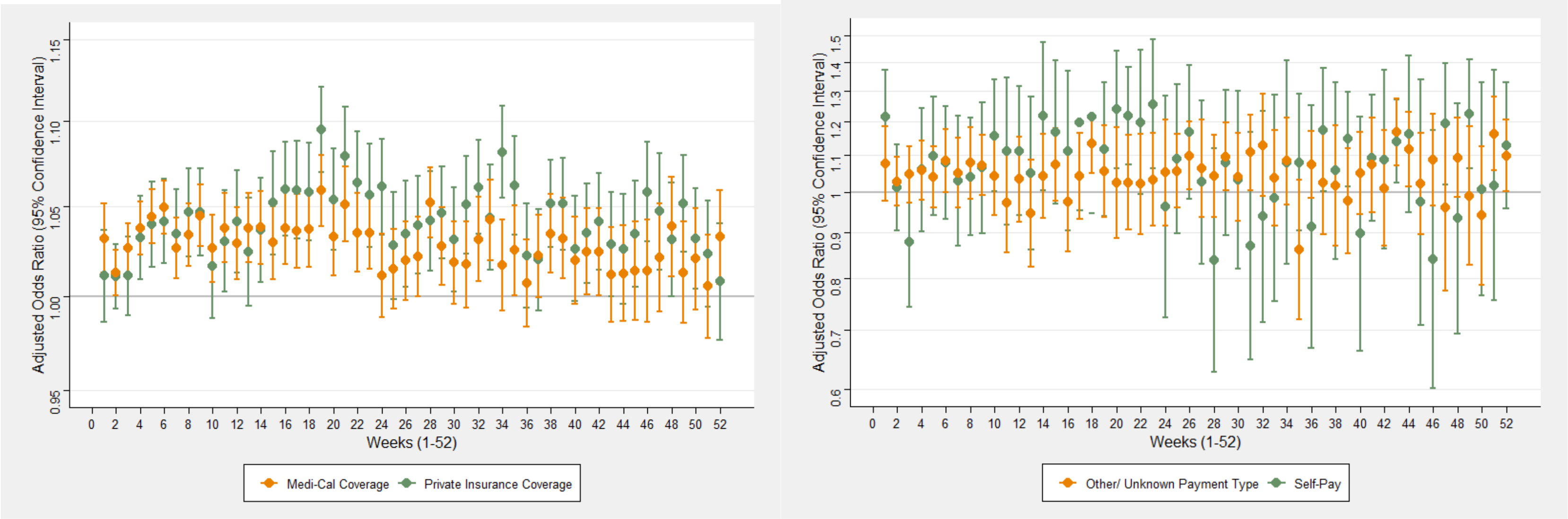

Models using a discrete time approach assessing the relationship between $PM_{2.5}$ exposure (increments of 5 $\mu g/m^3$) and time to all-cause ED visits during each week of the first year of life, where adjusted odds ratios and 95% confidence intervals are provided for the total population stratified by payment type for delivery (Left: Medi-Cal coverage in orange, private insurance coverage in green. Right: Other/ unknown payment type in orange, self-pay in green). These models were adjusted for parity, education, race and ethnicity, seasonality, and time-varying temperature. Y axes provided on the logarithmic scale.

© 2023 Teyton A et al. *JAMA Network Open.*
