# Supplementary material for: Exposure to Air Pollution and Emergency Department Visits During the First Year of Life Among Preterm and Full-term Infants
Source: JAMA Netw Open. 2023 Feb 22;6(2):e230262. doi: 10.1001/jamanetworkopen.2023.0262 (PMC9947725; doi:10.1001/jamanetworkopen.2023.0262)
Supplement: Supplement 2. — Data Sharing Statement [file jamanetwopen-e230262-s002.pdf]

Data Sharing Statement

Teyton. Exposure to Air Pollution and Emergency Department Visits During the First Year of Life Among Preterm and Full-term Infants. *JAMA Netw Open*. Published February 22, 2023. doi:10.1001/jamanetworkopen.2023.0262

Data
Data available: No

Additional Information
Explanation for why data not available: Per agreement with the California Department of Public Health, data may not be shared. However, those interested may apply for a SOMI Scholar appointment.
